# USP25 stabilizes STAT6 to promote IL-4-induced macrophage M2 polarization and fibrosis

**DOI:** 10.7150/ijbs.99345

**Published:** 2025-01-01

**Authors:** Yahan Xu, Jing Liu, Jingzeng Wang, Jiayao Wang, Peixiang Lan, Tao Wang

**Affiliations:** 1 Department of Respiratory and Critical Care Medicine, National Clinical Research Center of Respiratory Disease, Key Laboratory of Pulmonary Diseases of Health Ministry, Tongji Hospital, Tongji Medical College, Huazhong University of Science and Technology, Wuhan, Hubei, China.; 2 Institute of Organ Transplantation, Tongji Hospital, Tongji Medical College, Huazhong University of Science and Technology; Key Laboratory of Organ Transplantation, Ministry of Education; NHC Key Laboratory of Organ Transplantation; Key Laboratory of Organ Transplantation, Chinese Academy of Medical Sciences, Wuhan, China.

## Abstract

As a leading cause of morbidity and mortality, fibrosis is the common pathway of various chronic inflammatory diseases in organs and causes death in a large number of patients. It can destroy the structure and function of organs and ultimately lead to organ failure, which is a major cause of disability and death in many diseases. However, the regulatory mechanism of organ fibrosis is not well clear and the lack of effective drugs and treatments, which seriously endangers human health and safety. In this study, we found that ubiquitin specific peptidases 25 (USP25) deficiency could protect mice from bleomycin (BLM)-induced pulmonary fibrosis and bile duct ligation (BDL)-induced liver fibrosis. Mechanistically, USP25 deficiency reduced the infiltration of M2 macrophages in the lungs and livers. USP25 inhibits signal transducer and activator of transcription 6 / peroxisome proliferator-activated receptor gamma (STAT6/PPAR-γ) signaling by reducing the K48 specific ubiquitination of STAT6, thereby promoting IL-4-induced M2 macrophages. Overall, our findings support that USP25 promotes the development of fibrosis by facilitating macrophage M2 polarization.

## Introduction

Organ fibrosis presents the demise of parenchymal cells instigated by a myriad of injurious factors. An aberrant escalation of collagen and fibronectin within the extracellular matrix (ECM) gradually supplants normal tissue, precipitating organ dysfunction or outright failure^1^. Alarmingly, nearly half of all mortalities stemming from diseases worldwide are intertwined with organ fibrosis, thereby posing a grave threat to human well-being and security. Presently, the management of certain organ fibrotic disorders such as pulmonary fibrosis, liver fibrosis, and renal fibrosis heavily relies upon organ transplant^2^. Regrettably, the armamentarium of drugs available for combating fibrotic ailments is scarce, with a majority there of exhibiting suboptimal efficacy. In essence, the treatment landscape for organ fibrosis is marred by substantial limitations, prompting an exigent quest for novel therapeutic targets.

As the essential regulators of organ fibrosis, macrophages undergo marked phenotypic and functional changes after organ injury^3^-^5^. Clinical single-cell sequencing results have also elucidated the heterogeneous nature of macrophages within human organs, revealing a pronounced discrepancy in their characteristics between fibrotic and non-fibrotic environments^6^. Macrophages, integral to the innate immune system, are ubiquitously distributed across all tissues and organs, where they serve as potent phagocytes capable of eliminating pathogens. These cells are capable of assuming distinct functional phenotypes in response to varying physiological or pathological stimuli, a process known as macrophage polarization^7^. Macrophages can be differentiated into two polarization states: the classical activation phenotype (M1) and the alternative activation phenotype (M2)^8^. The M1 phenotype is intricately linked to pro-inflammatory responses, while the M2 phenotype is pivotal in orchestrating anti-inflammatory responses^9^, ^10^. Investigations have demonstrated that in the course of idiopathic pulmonary fibrosis (IPF), macrophages within the lungs predominantly adopt the M2 phenotype rather than the M1 phenotype^11^-^13^, and the elimination of M2 macrophages has been shown to mitigate lung fibrosis^14^-^17^.

Ubiquitination represents a post-translational protein modification renowned for its fundamental role in protein degradation via the ubiquitin-proteasome system^18^. Noteworthy for its versatility, the ubiquitination of target proteins undergoes a reversible process, prominently governed by deubiquitinating enzymes that oversee the intricate reversal of ubiquitination modifications^19^. Deubiquitinating enzymes can be classified into 7 major families with 98 members, of which USP is the most numerous families among the deubiquitinating enzymes^5^. Deubiquitinating enzymes possess the capability to counteract the polyubiquitination of target proteins, thereby modulating the functional alterations induced by ubiquitin modifications. Furthermore, they exert influence over the degradation and activation of substrate proteins, thereby finely orchestrating the propagation of cellular signaling pathways associated with the substrate^20^. Such regulatory mechanisms play a pivotal role in shaping the landscape of innate immunity.

In our study, we delved into the role of USP25 in tissue fibrosis by establishing pulmonary fibrosis models and liver fibrosis models in USP25 knockout (USP25^-/-^) mice. Our results suggest that deletion of USP25 promotes a surge in K48-specific ubiquitination of STAT6, thereby impairing the STAT6/PPAR-γ signaling pathway. It blocks macrophage M2 polarization, suppresses the expression of genes associated with tissue repair and fibrosis, ultimately attenuates the progression of fibrosis.

## Materials and Methods

### Human samples

Lung tissues from patients with non-small cell lung cancer (n=9) and idiopathic pulmonary fibrosis (IPF) (n=9) were collected in the Tongji Hospital with informed consent. This study was granted by the Human Assurance Committee of Tongji Hospital.

### Animals

USP25 knockout mice were donated by the Institute of Organ Transplantation of Tongji Hospital. WT (C57BL/6) mice were purchased from GemPharmatech Co., Ltd. All animals were housed in the specific pathogen-free animal facility at Tongji Hospital, with a 12:12h light-dark cycle. All animals had ad libitum access to food and water. The Animal Care and Use Committee of Tongji Hospital approved all procedures involving the mice (TJ-IRB20210351). Both male and female mice were used in all experiments.

### BLM induction of pulmonary fibrosis

WT and USP25^-/-^ mice (7-9 weeks old) were randomized to BLM-induced pulmonary fibrotic mice group or saline group. Mice in the BLM-induced pulmonary fibrosis group were anesthetized with 1% pentobarbital sodium and then administered 1.7mg/kg of BLM via airway injection. Mice receiving an equivalent volume of saline served as controls. Mice were sacrificed 21 days after BLM administration for analysis of pulmonary fibrosis.

### Preparation of bronchoalveolar lavage fluid (BALF)

BALF is collected by perfusion of the lungs with 0.7ml sterile saline, typically recovering about 0.5ml of BALF per mouse.

### Histopathological studies

Mice left lung and liver specimens were fixed in 4% neutral buffered formalin and embedded in paraffin. Routine histological analysis was performed on tissue sections by hematoxylin and eosin (H&E) staining. Sirius Scarlet and Masson staining were also used to evaluate paraffin sections for tissue lesions and fibrosis.

### Immunohistochemical staining

After autoclaving deparaffinized lung and liver sections in 10mM citric acid solution (pH 6.5) at 121℃ for 2min for antigen retrieval, and the tissues were blocked in 5% bovine serum. For immunostaining, the tissue section was probed with first antibody and then stained with the fluorescent secondary antibody of corresponding species.

### ELISA

ELISA kits include mouse IL-4 kit (Cat: EK0405, BOSTER, China), mouse IL-13 kit (Cat: EK0425, BOSTER, China), mouse TGF-β1 kit (Cat: RK00057, ABCLONAL, China). ELISA was performed according to the manufacturer's instructions.

### Bone marrow-derived macrophages (BMDMs) isolation and culture

Cut the femur and tibia of mice, flush the bone marrow, and resuspend it in PBS. The bone marrow cells were initially subjected to red blood cell lysis before being resuspended in DMEM culture medium supplemented with 10% fetal bovine serum, 1% penicillin/streptomycin, and 30ng/ml macrophage colony-stimulating factor (M-CSF). Subsequently, the cells were plated in cell culture dishes and incubated at 37°C, with the culture medium being refreshed every 2 days. After 7 days, the matured macrophages were co-cultured with IL-4 (10ng/ml) for the specified duration.

### Cell line and cell culture

The murine cell line NIH/3T3 was purchased from the Cell Bank of the Chinese Academy of Sciences (Shanghai, China). Cells were maintained in DMEM medium (Gibco), supplemented with 100 mg/mL of Streptomycin, 100 IU/mL of Penicillin and 10% fetal bovine serum (Cat: 164210-50, Pricella Life Science&Technology Co., Ltd) in an incubator at 37℃ and 5% CO_2_.

### Antibodies and reagents

Bleomycin hydrochloride (Cat: HY-17565A) was purchased from MedChemExpress (Monmouth Junction, NJ, USA). Recombinant murine IL-4 was obtained from PEPROTECH (Cat: 214-14). Recombinant Mouse M-CSF Protein was obtained from ABCLONAL (Cat: RP01216, ABCLONAL, China). Primary antibodies include anti-STAT6 antibody (Cat:380957, ZENBIO, China), anti PPAR-γ antibody (Cat: A11183, ABCLONAL, China), anti-USP25 antibody (Cat: ab187156, Abcam, USA), anti-Arg1 antibody (Cat: ab91279, Abcam, USA), anti-Ubi antibody (Cat: 134953, Abcam, USA), anti-Ub-k48 antibody (Cat: 140601, Abcam, USA). Cell culture mediums were purchased from Gibco. Protein A/G beads were purchased from Beyotime Biotechnology. Fetal bovine serum was purchased from Pricella Life Science&Technology Co., Ltd. USP25-PLvX Vector was purchased from Wuhan Augctbio. STAT6-PMYS Vector and HA-Ub-PMYS, HA-Ub-k48-PMYS Vector were self-made.

### Western blot analysis

Tissues and cultured cells were homogenized in RIPA lysis buffer and the protein concentration was measured using a BCA assay kit (Biyuntian, China). Protein samples of 40-80μg were denatured, separated using sodium dodecyl sulfate polyacrylamide gel electrophoresis (SDS-PAGE), and then transferred onto PVDF membranes. PVDF membranes were sealed with 5% skim milk prepared by TBST solution, and the primary antibodies were incubated for 16h. Membranes were then incubated in secondly antibodies for 2 hours and protein expression was detected by chemiluminescence.

### Immunoprecipitation

IP lysis buffer (Biyuntian, China) containing protease inhibitors extracted the total protein from cell lysates. To remove debris, lysates were centrifuged at 10,000×g for 10 min. Lysates were incubated with primary antibody overnight at 4℃ with continuous rotation. Immune-complexes were captured by incubating with Protein A/G beads overnight. Beads were then washed with PBS 5 times. The proteins were eluted with SDS loading buffer at 100 °C, followed by conducting immunoblotting to identify the proteins.

### Real-time quantitative polymerase chain reaction (RT-qPCR) analysis

Total ribonucleic acid (RNA) from cells or tissues was extracted using Total RNA Rapid Extraction Kit (Fastagen, Shanghai, China), and a high-capacity cDNA reverse transcription kit (TAKARA, Shiga, Japan) was used to reverse-transcribe total mRNA into cDNA according to the manufacturer's instructions. Then the SYBR Green kit (Takara) was used to quantify the expression levels of the target genes by quantitative polymerase chain reaction (qPCR) analysis. Each gene's relative amount was normalized to the quantity of β-actin, and the RT-qPCR analysis utilized the ΔΔ^-Ct^ algorithm. Primer sequences are shown in Table S1.

### Flow cytometry analysis

Cells isolated from tissues and BMDMs were stained with anti-mouse F4/80-APC (1: 100), CD206-FITC (1: 100) and CD11c-BV421 (1: 100) antibodies. After washes, the cells were analyzed by flow cytometry. Data analysis was performed using FlowJo 10.8.

### RNA sequencing analysis

After extracting total RNA from the samples, eukaryotic mRNA was enriched with oligo(dT)-conjugated magnetic beads. mRNA was cleaved into short fragments by adding fragmentation buffer. This mRNA was used as a template to synthesize first-strand cDNA using random hexamers, and second-strand cDNA was then synthesized by adding dNTPs, RNase H and DNA polymerase I. Next, the double-stranded cDNA was subjected to end repair, poly(A) tailing, ligation of sequencing junctions, purification and fragment selection using magnetic beads, and PCR amplification was finally conducted to obtain the library. Sequencing was performed after the library passed quality control. In our analysis, differentially expressed genes in biological duplicates were defined as those with |FoldChange| ≥ 2 and padj ≤ 0.05. Based on the results of the differential expression analysis and GO annotations, we performed GO enrichment analysis using clusterProfiler with p-adjust (FDR) < 0.05 as the threshold for filtering for significant enrichment. GO terms that met this criterion were defined as significantly enriched in the differentially expressed genes. Similar to the GO enrichment analysis process, we used clusterProfiler based on the results of differential expression analysis and KEGG annotations to identify significantly enriched KEGG pathways using p-adjust (FDR) < 0.05 as the threshold. RNA-seq data analysis was performed by Bioyigene Biotechnology, Wuhan, China.

### Statistical analysis

Comparative analyses between groups were conducted using the GraphPad Prism (version 8.0) software (GraphPad Software Inc., San Diego, CA, USA). Two experimental groups were compared using a Student's *t* test for paired data or a Student's* t* test with Welch's correction for unpaired data. For comparisons more than two groups, a one-way analysis of variance (ANOVA) was performed, followed by Bonferroni's correction. The data are presented as the mean ± SEM. The experiments followed the principle of randomization, and the data analysis was performed in a blinded manner whenever possible. P < 0.05 was considered statistically significant.

## Results

### USP25 is overexpressed in M2 macrophages and fibrotic tissues

**Figure 1:**
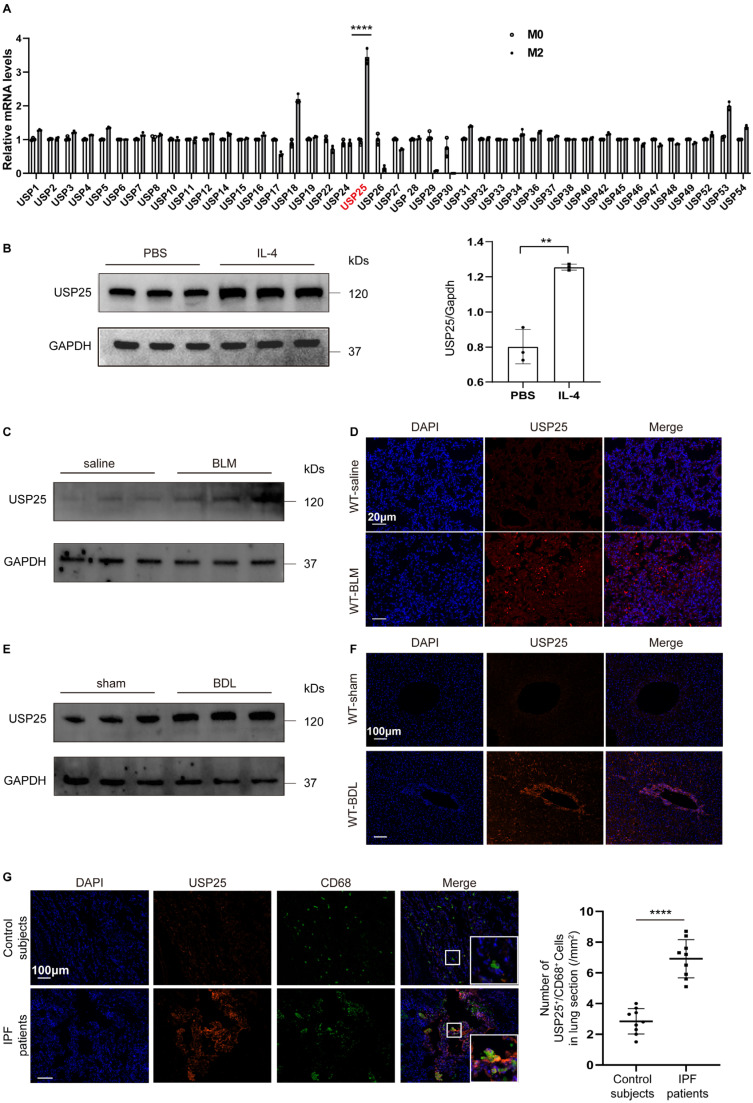
** USP25 show high expression in M2 macrophages and fibrotic tissues.** (A) Quantitative PCR analysis of USP family markers. (B) Western blot analysis of USP25 in homogenates from wild-type BMDM stimulated with IL-4 or vesicles. (C) Western blot analysis of USP25 in the lungs of mice following BLM induction. (D) Results for immunostaining of USP25 in BLM-induced lung sections. Scale bar, 20μm. (E) Western blot analysis of USP25 in the livers of mice following BDL induction. (F) Results for immunostaining of USP25 in BDL-induced liver sections. Scale bar, 100μm. (G) Results for co-immunostaining of USP25 and CD68 (macrophage marker) in the lung sections from patients with IPF and healthy subjects. Scale bar, 100μm. A total of nine IPF patients and nine control subjects were analyzed. Scatter plot indicates the USP25^+^/CD68^+^ cell count (numbers/mm^2^) in the lung sections from IPF patients and control subjects; each dot represents a patient. BLM, bleomycin; BDL, bile duct ligation; IPF, idiopathic pulmonary fibrosis. ****P < 0.0001

Previous studies have shown a close association between deubiquitinating enzymes and macrophage polarization^21^-^24^. In order to further determine the relationship between the USP family and macrophage polarization, we compared the expression of different USP molecules in M0 and M2 macrophages. First, BMDMs were extracted from wild-type (WT) mice and stimulated with IL-4 on day 7 to obtain M2 macrophages (Figure S1). Subsequent qPCR analysis found that USP25 was expressed at higher levels in M2 macrophages compared to other members of the USP family (Figure 1A). To further confirm this, we compared the protein levels of USP25 between M0 and M2 macrophages, and found the same trend (Figure 1B). The infiltration of M2 macrophages is closely related to the occurrence of tissue fibrosis^3^, ^25^, ^26^. Next, we examined USP25 expression in the lungs following BLM-induced pulmonary fibrosis and in the livers following BDL-induced liver fibrosis in animal models. Western blot and immunostaining showed that compared to normal tissues, USP25 was highly expressed in lung fibrotic areas (Figure 1 C, D) and liver fibrotic areas (Figure 1 E, F). Besides, we examined the expression of USP25 and macrophages (marked CD68) in fibrotic lungs of idiopathic pulmonary fibrosis (IPF) patients, and immunofluorescence showed that USP25 was highly expressed in fibrotic tissues and co-localized mainly with macrophages (Figure 1G). These results suggest that there may be a close relationship between USP25 and M2 macrophage mediated-tissue fibrosis.

### USP25 deficiency protects mice against BLM-induced lung injury and fibrosis

**Figure 2:**
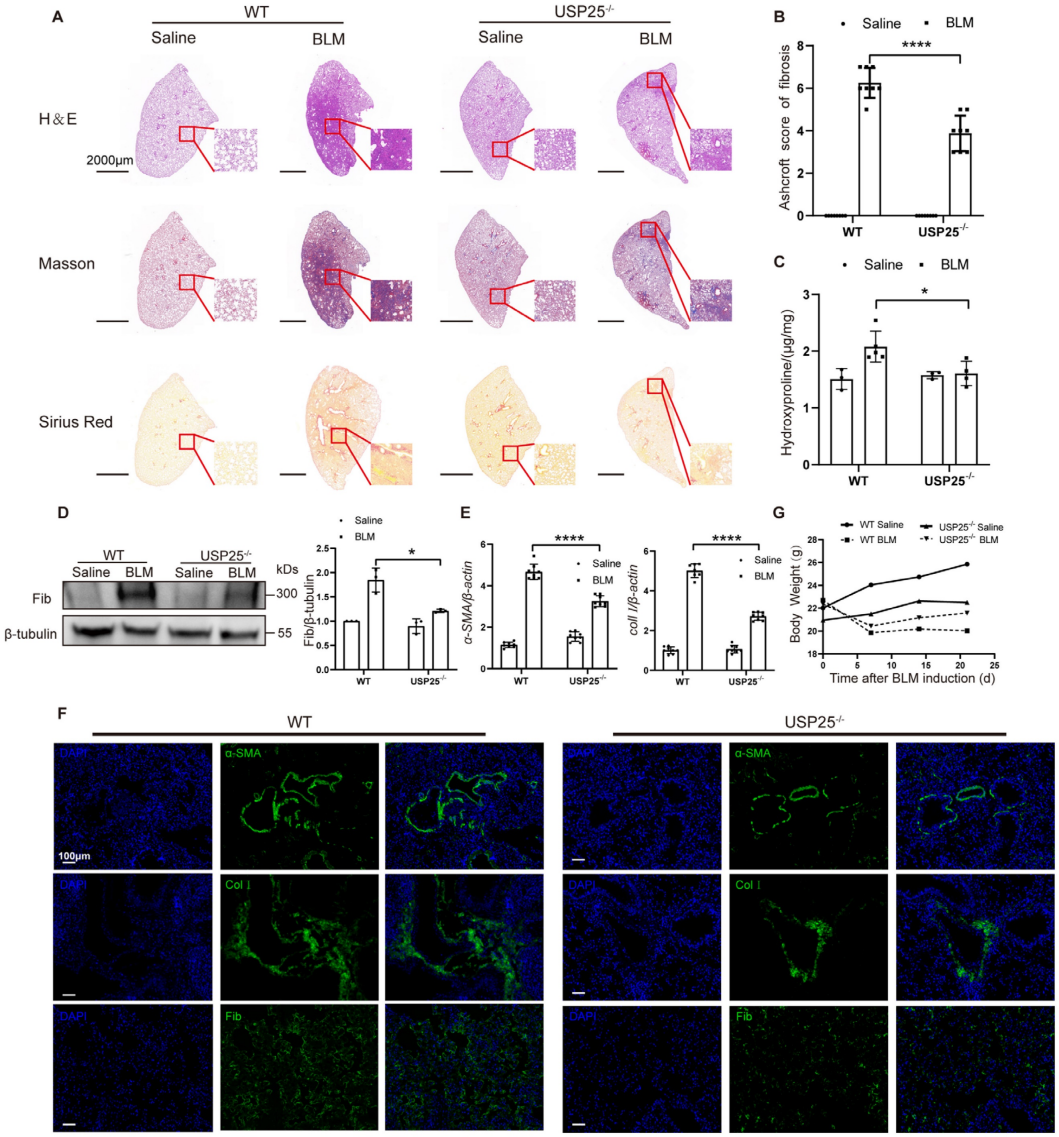
** USP25 deficiency protects mice against BLM-induced lung injury and fibrosis.** (A) Histological analysis of the severity of lung fibrosis in mice after BLM induction. Representative results for H&E (top), Masson (center), and Sirius red (bottom) staining. Scale bar, 2000μm. (B) Bar graph showing the semiquantitative Ashcroft scores for the severity of fibrosis. (C) Bar graph showing the quantification of hydroxyproline content in the lungs of mice after BLM induction. (D) Western blot analysis of the fibrotic marker fibronectin. (E) RT-qPCR analysis of *α-SMA* and *collagen Ⅰ*. (F) Results for immunostaining of the fibrotic markers α-SMA, collagen Ⅰ and fibronectin in BLM-induced lung sections. Scale bar, 100μm. (G) Body weight changes during the course of BLM-induced fibrosis. BLM, bleomycin; Col I, collagen I; Fib, fibronectin. The data are represented as the means ± SD. *p < 0.05 and ****P < 0.0001.

To further determine the role of USP25 in tissue fibrosis, we used USP25 knockout mice (USP25^-/-^ mice) (Figure S2). First, we induced a mouse pulmonary fibrosis model through BLM^17^, ^27^. Pulmonary fibrosis assessments were performed on WT and USP25^-/-^ mice after 21 days of BLM induction. Significantly attenuated lung injury and fibrosis as show in USP25^-/-^ mice by the H&E, Sirius red, and Masson's staining (Figure 2A). The lower Ashcroft score also demonstrated a significant reduction in the lung fibrosis in USP25^-/-^ mice (Figure 2B). We also measured the expression of hydroxyproline in the lung tissue homogenates, which is a marker associated with the severity of fibrosis. We found that the level of hydroxyproline in the lungs of USP25^-/-^ mice was lower than WT mice (Figure 2C). Consistent with these observations, Western blot analysis showed that the expression of fibronectin, a fibrosis marker, in the lungs of USP25^-/-^ mice was reduced significantly (Figure 2D). Furthermore, RT-qPCR analysis also indicated that the expression of fibrosis-related molecules α-SMA and collagen Ⅰ in the lungs of USP25^-/-^ mice was significantly decreased (Figure 2E). Immunostaining of lung sections also demonstrated a significant reduction in the expression of fibronectin, α-SMA and collagen Ⅰ in USP25^-/-^ mice (Figure 2 F). 7 days after BLM induction, both WT and USP25^-/-^ mice manifested a significant weight loss, which is a common phenotype usually associated with pulmonary fibrosis. However, the USP25^-/-^ mice showed a gradual weight increase on days 14 and 21 after BLM induction, whereas the WT mice continued to show a sustained decrease in weight (Figure 2G). Overall, these results indicated that the deficiency of USP25 could protect mice from BLM-induced lung injury and fibrosis.

### USP25 deficiency protects mice against liver fibrosis

**Figure 3:**
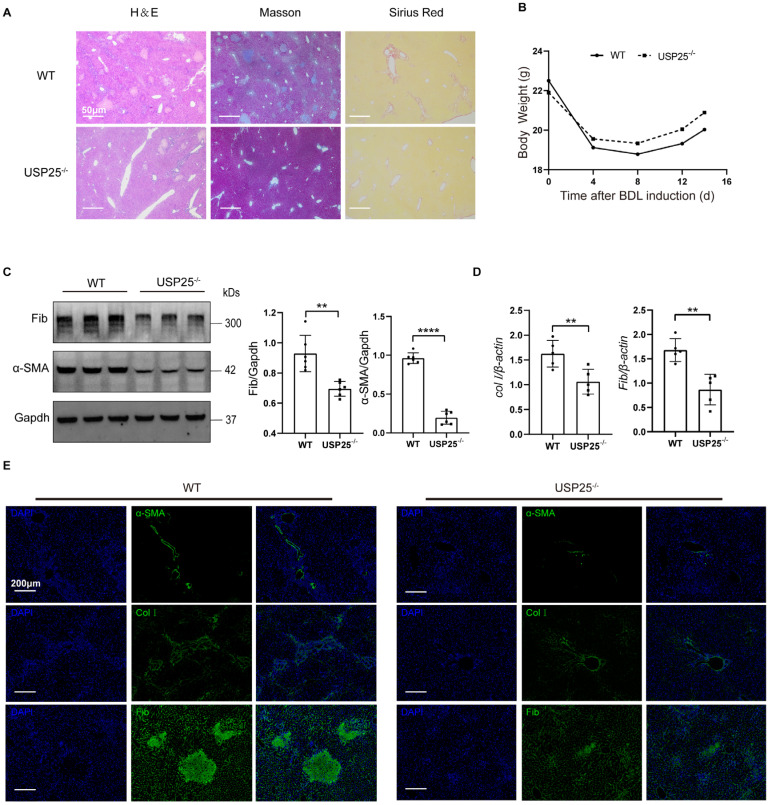
** USP25 deficiency attenuate liver fibrosis.** (A) Histological analysis of the severity of liver fibrosis in mice after BDL induction. Representative results for H&E (left), Masson (center), and Sirius red (right) staining. Scale bar, 50 μm. (B) Body weight changes during the course of BDL-induced liver fibrosis. (C) Western blot analysis of fibronectin and α-SMA in the liver homogenates. (D) RT-qPCR analysis of *collagen I* and *fibronectin*. (E) Results for immunostaining of the fibrotic markers α-SMA, collagen I and fibronectin in BDL-induced liver sections. Scale bar, 200μm. BDL, bile duct ligation; Col I, collagen I; Fib, fibronectin. The data are represented as the means ± SD.**p < 0.01 and ****p < 0.0001.

To further confirm the effect of USP25 on tissue repair and fibrosis, we established a mouse liver fibrosis model by BDL for 14 days. Pathological results showed that the degree of liver fibrosis in USP25^-/-^ mice was significantly reduced compared to WT mice (Figure 3A). The weight loss of USP25^-/-^ mice was significantly lower than that of WT mice (Figure 3B). Western blot analysis of liver homogenates revealed a significant reduction in the expression of fibronectin and α-SMA in USP25^-/-^ mice as compared to the WT mice (Figure 3C). RT-qPCR analysis also indicated that the expression of collagen Ⅰ and fibronectin in the livers of USP25^-/-^ mice was significantly decreased (Figure 3D). Furthermore, immunostaining of liver sections also demonstrated a same trend (Figure 3E). Collectively, USP25 deficiency can protect against liver fibrosis.

### USP25 deficiency attenuates macrophage M2 polarization

**Figure 4:**
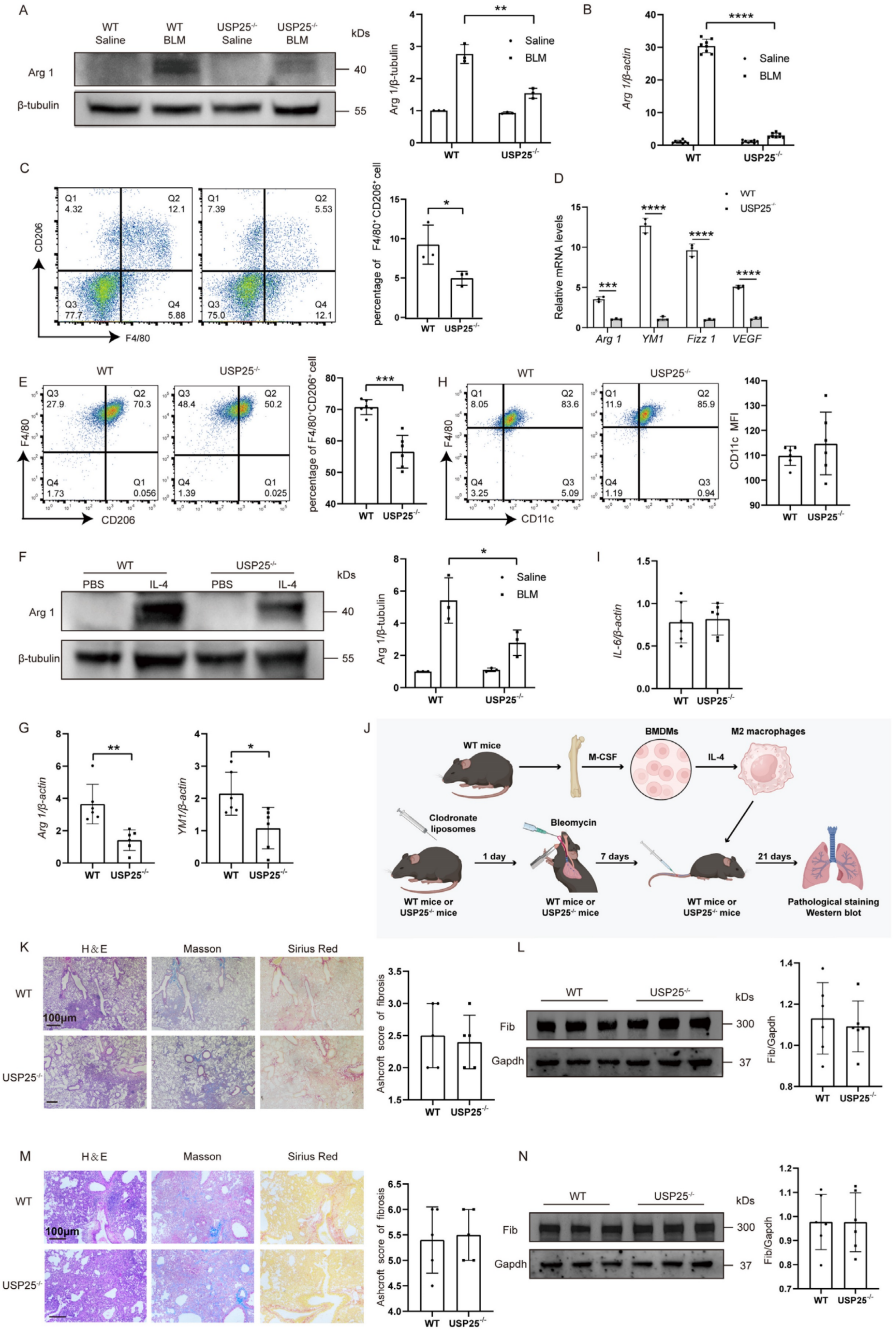
** USP25 deficiency attenuates M2 polarization in macrophages.** (A) Western blot analysis of Arg 1 in the lung homogenates. (B) RT-qPCR results for analysis of *Arg 1* expression in the lung. (C) Flow cytometry analysis of M2 macrophages in liver-derived macrophages. (D) RT-qPCR for analysis of *Arg 1*, *YM1*, *Fizz1*, and *VEGF* expression in liver derived macrophages. (E) Flow cytometry analysis of CD206 expression in BMDMs following IL-4 stimulation. (F) Western blot analysis of Arg 1 in BMDMs after IL-4 induction. (G) RT-qPCR for analysis of *Arg 1* and *YM1* expression in the BMDMs after IL-4 induction. (H) Flow cytometry analysis of CD11c expression in BMDMs following LPS stimulation. (I) RT-qPCR analysis of *IL-6* expression in the BMDMs after LPS induction. (J) The schematic representation for the macrophage adoptive transfer experiments. IL-4-stimulated WT M2 BMDMs were selectively transferred into both WT and USP25^-/-^ mice that had been pretreated with clodronate liposomes, via tail vein injection on the seventh day post BLM induction. (K) The severity of pulmonary fibrosis in WT and USP25^-/-^ mice after depletion of macrophages. Right: a bar graph figure showing the semiquantitative Ashcroft scores for the severity of pulmonary fibrosis. (L) Western blot analysis of fibronectin in the lungs of macrophage-depleted mice after BLM induction. (M) Results for adoptive transfer of WT macrophages into WT and USP25^-/-^ mice following BLM induction. Right: the semiquantitative Ashcroft scores relevant to the severity of fibrosis. (N) Western blot analysis of fibronectin expression. Arg 1, arginase 1; YM1, chitinase 3-like 3; Fizz1, found in inflammatory zone; VEGF, vascular endothelial growth factor; BMDMs, bone marrow-derived macrophages; BLM, bleomycin; Fib, fibronectin. The data are represented as the means ± SD. *p < 0.05, **p < 0.01, ***p < 0.001 and ****P < 0.0001.

To further determine the correlation between USP25-mediated tissue fibrosis and M2 macrophage polarization, we next tested M2 macrophage polarization related indicators in pulmonary and liver fibrosis models. In a pulmonary fibrosis model, Western blot (Figure 4A) and RT-qPCR analysis (Figure 4B) found that expression of M2-labeled Arg1 in USP25^-/-^ mice was significantly reduced compared to WT mice. In the liver fibrosis model, we extracted hepatic macrophages for flow cytometry and found that the proportion of M2 macrophages of USP25^-/-^ mice was significantly lower than that of WT mice (Figure 4C). At the same time, RT-qPCR results of hepatic macrophages showed that the expression levels of Arg1, YM1, Fizz1 and VEGF were significantly reduced in USP25^-/-^ mice (Figure 4D). To further determine that USP25 regulates the polarization of M2 macrophages, we extracted BMDMs from WT and USP25^-/-^ mice and then stimulated with IL-4. Flow cytometry results showed that the percentage of M2 macrophages (F4/80^+^CD206^+^) induced from WT BMDMs was significantly higher than that from USP25^-/-^ BMDMs (Figure 4E). Western blot analysis showed that the expression of Arg1 was reduced in USP25^-/-^ BMDMs compared to WT BMDMs (Figure 4F). The RT-qPCR analysis of Arg1 and another M2 marker, YM1, also showed a consistent trend (Figure 4G). Notably, flow cytometry and RT-qPCR demonstrated that USP25 deficiency did not affect the induction of M1 macrophages (Figure 4H) and the expression of IL-6 (Figure 4I).

Based on the above results, we speculate that USP25 deficiency protected mice against BLM-induced lung fibrosis and BDL-induced liver fibrosis due to the repression of macrophage M2 polarization. To further elucidate this issue, we used clodronate liposomes to deplete macrophages in the lungs. Clodronate liposomes was administered by tail vein combined with trachea. Pulmonary fibrosis was induced by BLM 1 day after depletion of macrophages (Figure 4J). In concordance with our predictions, the extent of fibrosis within the lungs of USP25^-/-^ mice was found to be equivalent to that observed in WT mice after depleting of macrophages (Figure 4K and 4L). Subsequently, BMDMs in WT mice were induced by IL-4 to differentiate into M2 macrophages. 1×10^6^ M2 macrophages was adoptively transferred via the tail vein into the mice previously treated with clodronate liposome (Figure 4J). Transfusion of WT M2 macrophages was found to significantly attenuate the protective effect of USP25 deficiency (Figure 4M and 4N). In summary, these results indicated that USP25 deficiency protected mice from tissue injury and fibrosis by inhibiting macrophage M2 polarization.

### USP25 deficiency inhibition of TGF-β secretion by M2 macrophages

**Figure 5:**
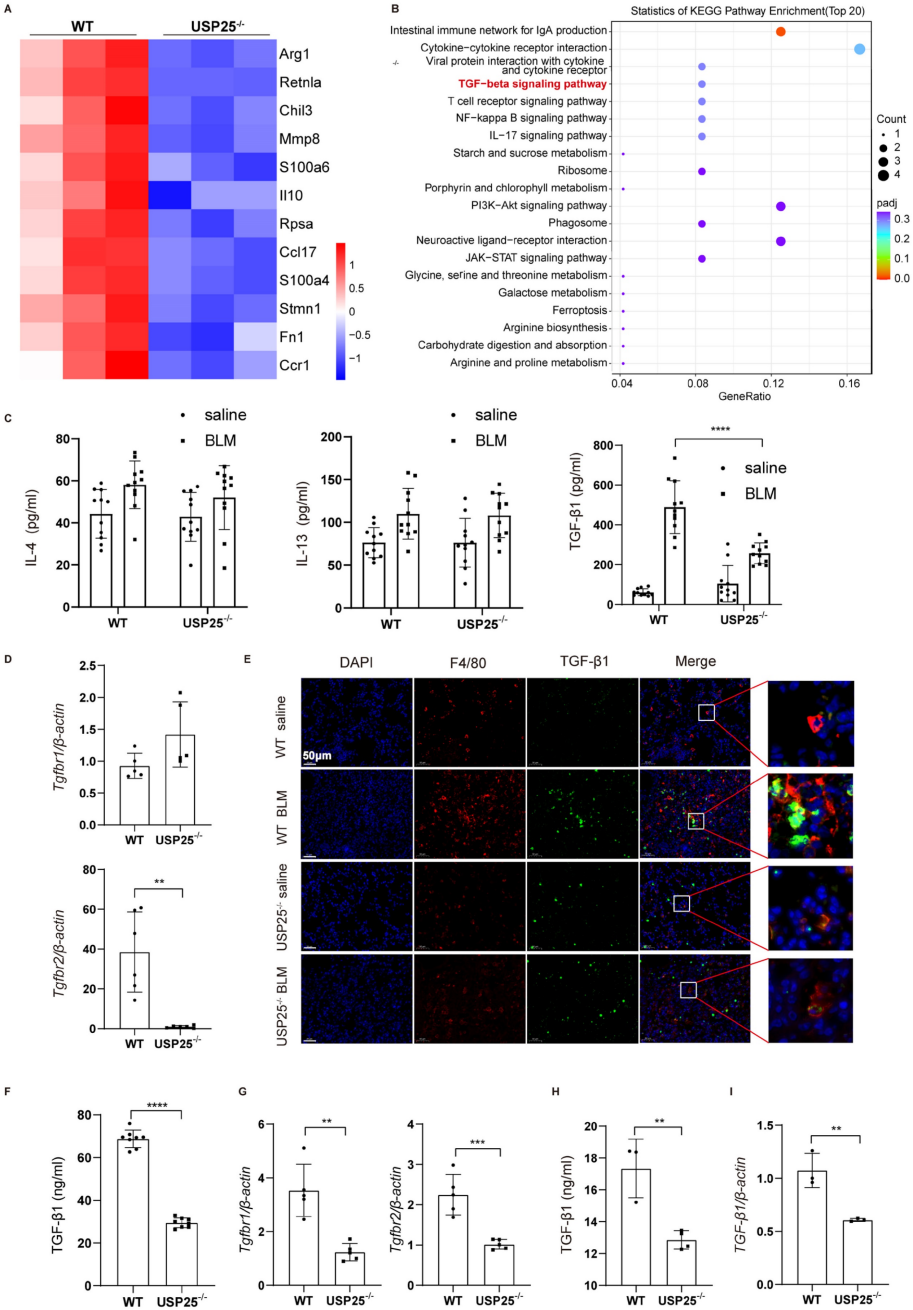
** USP25 deficiency suppresses M2 macrophages TGF-β signaling.** (A) RNA seq analysis results of WT and USP25^-/-^ mice IL-4 stimulated BMDMs. Differential gene clustering heat map. (B) RNA seq analysis results of alveolar macrophages from WT and USP25^-/-^ mice after BLM induction. KEGG pathway analysis bubble diagram. (C) ELISA results for IL-4, IL-13 and TGF-β1 levels in the BALF. (D) RT-qPCR results for *Tgfbr1* and *Tgfbr2* expression in the lungs after BLM induction. (E) Co-immunostaining of F4/80 and TGF-β1 in the lung sections. Scale bar, 50 μm. (F) ELISA results for TGF-β1 level in the mice serum after BDL induction. (G) RT-qPCR results for *Tgfbr1* and *Tgfbr2* expression in the livers after BDL induction. (H) ELISA results for TGF-β1 level in the cell culture medium. (I) RT-qPCR results for *TGF-β1* expression in the BMDMs stimulated with IL-4. BALF, bronchoalveolar lavage fluid; BLM, bleomycin; TGF-β1, transforming growth factor β1. The data are represented as the means ± SD. **p < 0.01, ***p < 0.001 and ****P < 0.0001.

To explore how M2 macrophages play a role in USP25-induced tissue fibrosis, we extracted BMDMs from WT mice and USP25^-/-^ mice, stimulated with IL-4 to obtain M2 macrophages, and then subjected to RNA sequencing analysis. We identified 732 differentially expressed genes, among which 304 genes were downregulated in the USP25^-/-^ group (figure S3). Within this genetic cohort, M2 macrophage-associated genes, including Arg1, Retnla, and Chil3, were significantly reduced in USP 25-deficient BMDMs (Figure 5A). Since IL-4, IL13 and TGF-β1 play an important role in the progression of fibrosis, we further analyzed these signaling pathways in these two groups. The KEGG pathway enrichment analysis results indicated significant differences in the TGF-β signaling pathway rather than IL-4 or IL-13 (Figure 5B). Next, levels of IL-4, IL-13 and TGF-β1 in BALF of WT and USP25^-/-^mice after BLM-induced pulmonary fibrosis models were measured by ELISA. It was also found that compared with WT mice, there were no significant statistical differences in IL-4 and IL-13 in USP25^-/-^ mice, while TGF-β1 was significantly reduced (Figure 5C). Additionally, Tgfbr2, a major TGF-β1 receptor, showed significantly lower mRNA expression levels in the lungs of USP25^-/-^ mice than in WT mice after BLM induction (Figure 5D). Another major TGF-β1 receptor, Tgfbr1, did not show significant differences between USP25^-/-^ and WT mice (Figure 5D). Co-immunostaining of lung sections from BLM-challenged mice showed that macrophages (F4/80^+^) were the main cells secreting TGF-β1(Figure 5E). Besides, USP25^-/-^ mice exhibited significantly fewer TGF-β1^+^F4/80^+^ cells after BLM induction (Figure 5E). Similarly, in BDL-induced liver fibrosis model, we measured TGF-β1 levels in mouse serum by ELISA. TGF-β1 in USP25^-/-^ mice was significantly lower than that in WT mice (Figure 5F). RT-qPCR analysis also indicated that the expression of Tgfbr1 and Tgfbr2 in the livers of USP25^-/-^ mice was significantly decreased (Figure 5G). *In vitro* experiments, we used ELISA to detect the level of TGF-β1 in the culture medium of BMDMs stimulated with IL-4 and found that compared to WT BMDMs, the level of TGF-β1 in USP25^-/-^ BMDMs was reduced (Figure 5H). Similar results were also observed at the mRNA level by RT-qPCR (Figure 5I). In a word, our data suggested that USP25 deficiency weakened the ability of macrophages to secrete TGF-β1 and inhibited tissue fibrosis.

### USP25 inhibits K48 ubiquitination of STAT6 to enhance STAT6/PPAR-γ signaling in macrophages

**Figure 6:**
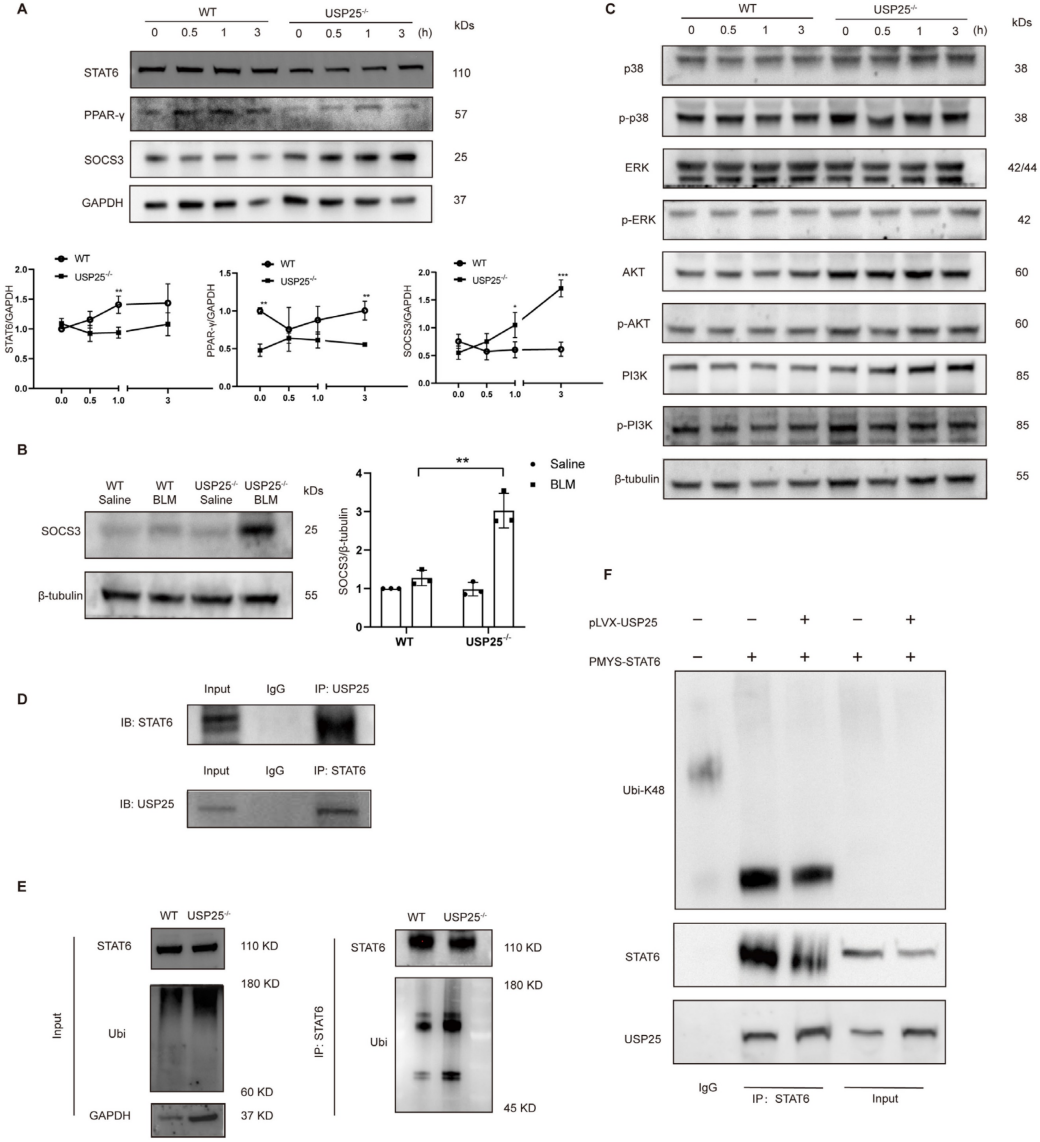
** USP25 inhibit K48 ubiquitination of STAT6 to enhance STAT6/PPAR-γ signaling in macrophages.** (A) Upper panel: representative Western blot results for STAT6, PPAR-γ and SOCS3 at different time points stimulated with IL-4. Lower panel: figures showing the data with three mice analyzed. (B) Western blot analysis of SOCS3 in the lung homogenates. (C) USP25 did not affect MAPK (p38 and ERK), Akt and PI3K signaling. (D) Upper panel: immunoprecipitation of proteins using USP25 antibody and immunoblotting analysis using STAT6 antibody. Lower panel: immunoprecipitation of proteins using STAT6 antibody and immunoblotting analysis using USP25 antibody. (E) BMDMs of WT and USP25^-/-^ were treated with MG132 (10 μM) for 6 h before lysis, followed by stimulation of IL-4 (10 ng/mL) for 30 min. Proteins were immunoprecipitated with STAT6 antibody and analyzed by immunoblotting with the indicated antibodies. (F) NIH/3T3 cells were transfected with plasmids encoding the indicated constructs. Proteins were immunoprecipitated with anti-STAT6 antibody and analyzed by immunoblotting with the indicated antibodies. IP, immunoprecipitation; STAT6, signal transducer and activator of transcription 6; PPAR-γ, peroxisome proliferator-activated receptor gamma; SOCS3, suppressor of cytokine signaling 3. *p < 0.05 and **p < 0.01.

To dissect the mechanisms by which USP25 deficiency represses M2 polarization in macrophages, we investigated the STAT6/PPAR-γ signaling pathway, which is an important pathway for inducing macrophage M2 polarization^28^-^30^. There were no significant differences in the levels of STAT6 and PPAR-γ between WT and USP25^-/-^ BMDMs (Figure 6A). However, STAT6 was highly expressed in WT BMDM after 30 minutes of IL-4 stimulation, and its expression continued to increase as the stimulation time extended (Figure 6A).

Under the same conditions, changes in STAT6 and PPAR-γ were not significant in USP25^-/-^ BMDMs (Figure 6A). Subsequently, we also examined the expression of suppressors of cytokine signaling 3 (SOCS3), an inhibitor of STAT6. The results showed that WT BMDMs maintained low levels of SOCS3 under IL-4 stimulation. In sharp contrast, USP25^-/-^ BMDMs exhibited time-dependent high expression of SOCS3 in response to IL-4 stimulation (Figure 6A). Western blot analysis of lung homogenates revealed that SOCS3 was expressed at low levels in the control group and slightly increased after BLM induction (Figure 6B). However, in USP25^-/-^ mice, the expression of SOCS3 was significantly elevated (Figure 6B). Additionally, signaling pathways such as p38, ERK, AKT, and PI3K are also involved in macrophage M2 polarization, but the absence of USP25 seemed to have no effect on these signaling pathways (Figure 6C).

Based on the above results, we next explored the mechanism by which USP25 regulates the STAT6/PPAR-γ signaling pathway. We found that after co-immunoprecipitation with USP25 antibody, STAT6 was detected by Western blot. Similarly, USP25 was detected by Western blot after co-immunoprecipitation with STAT6 antibody (Figure 6D). Combined with the downregulation of STAT6 protein levels after USP25 knockout (Figure 6A), these results suggested that US25 can interact with STAT6 in macrophages. USP25 is a deubiquitinase which can regulate intracellular signal transduction by altering the ubiquitination state of proteins, we assessed the ability of USP25 to regulate STAT6 ubiquitination. We treated BMDMs with MG132 for 6 hours to block the degradation of ubiquitinated proteins by the proteasome, and then treated with IL-4 for 30 minutes. Immunoprecipitation assay was used to detect the level of ubiquitination of STAT6 in BMDMs, and the results showed that knockdown of USP25 led to an increase in STAT6 ubiquitination (Figure 6E). USP25 is a multifunctional deubiquitinase that can regulate various types of ubiquitination modifications, including K48 and K63. Since K48 specific ubiquitination is a key signal for protein degradation mediated by the proteasome and is the most common type of ubiquitination. In further experiments, we transiently transfected STAT6 and USP25 into the NIH/3T3 cell line separately and detected the K48 specific ubiquitination level of total proteins. We found that the K48 specific ubiquitination level of total proteins in NIH/3T3 cells transfected with the USP25 expression vector was significantly reduced (Figure 6F). Additionally, in NIH/3T3 cells co-transfected with STAT6 and USP25 overexpression vectors, we observed that the K48 specific ubiquitination of STAT6 was decreased in the presence of USP25 (Figure 6F). In summary, these findings suggested that USP25 protected STAT6 from degradation through K48 specific ubiquitination.

## Discussion

In this study, we found that the absence of USP25 improved pulmonary and liver fibrosis in mice. Delving into the mechanistic underpinnings, we observed that USP25 deficiency increases the ubiquitination of STAT6 in macrophages, thereby blocking the STAT6/PPAR-γ signaling pathway, and inhibiting the polarization of macrophages towards the M2 phenotype. M2 macrophages from USP25^-/-^ mice reduce the ability to secrete TGF-β1 and inhibit tissue fibrosis. Collectively, these findings underscore the promise of targeting USP25 as a viable approach in the realm of organ fibrosis prevention and treatment.

Macrophages exhibit a remarkable degree of heterogeneity and adaptability. The differentiation and activation process, by which macrophages assume specific functional states in response to environmental cues and stimuli, is known as macrophage polarization^31^. Depending on the degree of polarization and the neighboring microenvironment, macrophages exert a Janus-faced influence, simultaneously promoting and impeding fibrotic progression^5^. Macrophages can secrete matrix metalloproteinases to degrade the ECM, thereby reducing the deposition of collagen^32^. In addition, macrophages can also secrete various pro-fibrotic cytokines and chemokines to promote the development of fibrosis^33^, ^34^. M1 macrophages play a key role in promoting inflammatory responses, while M2 macrophages contribute to anti-inflammatory responses and tissue remodeling^9^, ^35^. M2 macrophages are indispensable participants in tissue repair, capable of secreting inflammatory inhibitory factors and promoting epithelial cells to produce ECM. Studies have indicated that during the progression of IPF, M2 macrophages are ubiquitous in the lungs^12^, ^36^, ^37^.

The TGF-β signaling pathway exerts a pivotal influence in modulating cellular proliferation and differentiation, thereby impacting diverse cellular entities within the fibrotic microenvironment, including macrophages, epithelial cells, and endothelial cells^38^. This pathway is considered a major regulator of ECM remodeling. TGF-β1 can mediate epithelial-mesenchymal transition (EMT) of alveolar epithelial cells, activate interstitial cells to synthesize ECM, and provide a favorable microenvironment for the occurrence and development of IPF^39^, ^40^. During the development of liver fibrosis, the TGF-β signaling pathway can stimulate the production of ECM and inhibit its degradation, thereby accelerating liver fibrosis^41^. In our study, USP25 deficiency attenuated BLM-induced TGF-β1 in the BALF by onefold. RNA sequencing also demonstrated a large difference in the TGF-β signaling pathway in alveolar macrophages from WT and USP25^-/-^ mice. It is noteworthy, however, in addition to macrophages, positive staining of TGF-β1 was also observed in other cell types. Based on previous studies, they could be alveolar epithelial cells and mesenchymal cells, which will be addressed in our future studies.

Ubiquitination and deubiquitination represent pivotal regulatory mechanisms influencing the polarization of macrophages. USP19, functioning as an inflammatory pivot, has been demonstrated to suppress inflammatory responses and to facilitate the polarization of M2 macrophages^21^. TRAF3 emerges as a crucial mediator in M2 polarization, orchestrating this process through the regulation of STAT6 K450 ubiquitination within macrophages^23^. USP10 catalyzes the deubiquitination of NLRP7, a molecular event that propels the polarization of pro-tumorigenic M2-like macrophages^22^. The E3 ubiquitin ligase TRIM21 has been demonstrated to facilitate the ubiquitination of Sohlh2, thereby impeding macrophage M2 polarization^24^. In this study, we demonstrated that USP25 also participated in and regulated macrophage M2 polarization, providing a new and potential therapeutic target for the treatment of fibrosis. STAT6 emerged as a pivotal transcription factor in the orchestration of macrophage M2 polarization^29^, ^42^, while PPAR-γ stood as an indispensable component in the maturation of M2 macrophages^43^. IL-4 and IL-13 have the capability to directly trigger the macrophage M2 polarization orchestrated by STAT6. Moreover, the suppression of SOCS activity has been noted to amplify the STAT6/PPAR-γ signaling pathway, thereby fostering macrophage M2 polarization^44^. Our research found that the deficiency of USP25 significantly enhanced the activity of SOCS3 and inhibited the expression of STAT6 and PPAR-γ induced by IL-4. Furthermore, we observed USP25 enhanced the K48 ubiquitination of STAT6.

It is undeniable that our study has certain limitations. In the study, we used mice with global knockout of the USP25. Although the deficiency of USP25 inhibited macrophage M2 polarization, thus reducing fibrosis, we anticipate that other cell types, including epithelial cells, fibroblasts, T lymphocytes, mast cells, etc, might also be affected by the deficiency of USP25. Subsequent studies can be conducted more rigorously by constructing macrophage-specific knockout USP25 mice.

Our study revealed that the deletion of USP25 can inhibit the STAT6/PPAR-γ signaling pathway by enhancing the ubiquitination of STAT6, thereby affecting macrophage M2 polarization, ultimately alleviating BLM-induced pulmonary fibrosis and bile duct ligation-induced liver fibrosis.

In conclusion, although the contributions to fibrosis reduction mediated by USP25 deficiency from other cell subpopulations besides macrophages cannot be excluded, our data still robustly support that USP25 may be a feasible target for the clinical treatment of organ fibrosis.

## Supplementary Material

Supplementary figures and table.

## Figures and Tables

**Figure 7:**
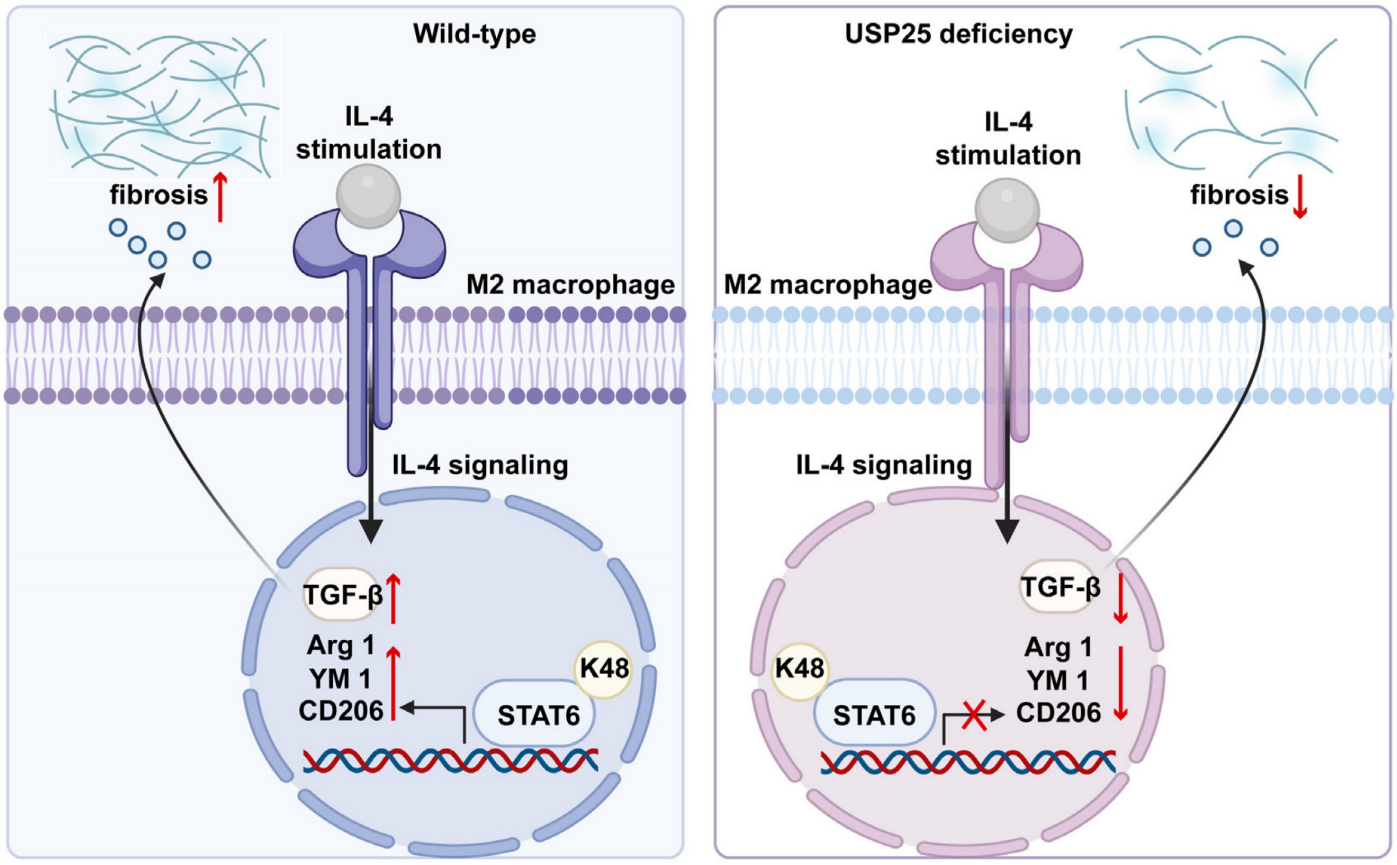
Schematic mechanism of USP25 deficiency inhibits macrophage M2 polarization and the development of fibrosis.

## Abbreviations

USP25: ubiquitin specific peptidases 25
BLM: bleomycin
BDL: bile duct ligation
STAT6: signal transducer and activator of transcription 6
PPAR-γ: peroxisome proliferator-activated receptor gamma
ECM: extracellular matrix
EMT: epithelial-mesenchymal transition
IPF: idiopathic pulmonary fibrosis
H&E: hematoxylin and eosin
BALF: bronchoalveolar lavage fluid
BMDMs: bone marrow-derived macrophages
M-CSF: macrophage colony-stimulating factor
WT: wild type
SOCS3: suppressors of cytokine signaling 3

## References

[1] Karsdal MA, Nielsen SH, Leeming DJ, Langholm LL, Nielsen MJ, Manon-Jensen T (2017). The good and the bad collagens of fibrosis - Their role in signaling and organ function. Advanced drug delivery reviews.

[2] Henderson NC, Rieder F, Wynn TA (2020). Fibrosis: from mechanisms to medicines. Nature.

[3] Wynn TA, Vannella KM (2016). Macrophages in Tissue Repair, Regeneration, and Fibrosis. Immunity.

[4] Chen M, Menon MC, Wang W, Fu J, Yi Z, Sun Z (2023). HCK induces macrophage activation to promote renal inflammation and fibrosis via suppression of autophagy. Nature communications.

[5] Murray PJ, Wynn TA (2011). Protective and pathogenic functions of macrophage subsets. Nature reviews Immunology.

[6] Reyfman PA, Walter JM, Joshi N, Anekalla KR, McQuattie-Pimentel AC, Chiu S (2019). Single-Cell Transcriptomic Analysis of Human Lung Provides Insights into the Pathobiology of Pulmonary Fibrosis. Am J Respir Crit Care Med.

[7] Mantovani A, Sica A, Sozzani S, Allavena P, Vecchi A, Locati M (2004). The chemokine system in diverse forms of macrophage activation and polarization. Trends Immunol.

[8] Locati M, Curtale G, Mantovani A (2020). Diversity, Mechanisms, and Significance of Macrophage Plasticity. Annual review of pathology.

[9] Funes SC, Rios M, Escobar-Vera J, Kalergis AM (2018). Implications of macrophage polarization in autoimmunity. Immunology.

[10] Zhao Y, Chen S, Lan P, Wu C, Dou Y, Xiao X (2018). Macrophage subpopulations and their impact on chronic allograft rejection versus graft acceptance in a mouse heart transplant model. American journal of transplantation: official journal of the American Society of Transplantation and the American Society of Transplant Surgeons.

[11] Zhang L, Wang Y, Wu G, Xiong W, Gu W, Wang C-Y (2018). Macrophages: friend or foe in idiopathic pulmonary fibrosis?. Respiratory research.

[12] Cheng P, Li S, Chen H (2021). Macrophages in Lung Injury, Repair, and Fibrosis. Cells.

[13] Yao Y, Wang Y, Zhang Z, He L, Zhu J, Zhang M (2016). Chop Deficiency Protects Mice Against Bleomycin-induced Pulmonary Fibrosis by Attenuating M2 Macrophage Production. Molecular therapy: the journal of the American Society of Gene Therapy.

[14] Bonniaud P, Kolb M, Galt T, Robertson J, Robbins C, Stampfli M (2004). Smad3 null mice develop airspace enlargement and are resistant to TGF-beta-mediated pulmonary fibrosis. Journal of immunology (Baltimore, Md: 1950).

[15] Wang Y, Zhang L, Wu GR, Zhou Q, Yue H, Rao LZ (2021). MBD2 serves as a viable target against pulmonary fibrosis by inhibiting macrophage M2 program. Science advances.

[16] Chen Y, Wang T, Liang F, Han J, Lou Z, Yu Y (2024). Nicotinamide phosphoribosyltransferase prompts bleomycin-induced pulmonary fibrosis by driving macrophage M2 polarization in mice. Theranostics.

[17] Rao LZ, Wang Y, Zhang L, Wu G, Zhang L, Wang FX (2021). IL-24 deficiency protects mice against bleomycin-induced pulmonary fibrosis by repressing IL-4-induced M2 program in macrophages. Cell death and differentiation.

[18] Swatek KN, Usher JL, Kueck AF, Gladkova C, Mevissen TET, Pruneda JN (2019). Insights into ubiquitin chain architecture using Ub-clipping. Nature.

[19] Fang Y-Z, Jiang L, He Q, Cao J, Yang B (2023). Deubiquitination complex platform: A plausible mechanism for regulating the substrate specificity of deubiquitinating enzymes. Acta Pharm Sin B.

[20] Sun T, Liu Z, Yang Q (2020). The role of ubiquitination and deubiquitination in cancer metabolism. Mol Cancer.

[21] Liu T, Wang L, Liang P, Wang X, Liu Y, Cai J (2021). USP19 suppresses inflammation and promotes M2-like macrophage polarization by manipulating NLRP3 function via autophagy. Cellular & molecular immunology.

[22] Li B, Qi ZP, He DL, Chen ZH, Liu JY, Wong MW (2021). NLRP7 deubiquitination by USP10 promotes tumor progression and tumor-associated macrophage polarization in colorectal cancer. Journal of experimental & clinical cancer research: CR.

[23] Shi JH, Liu LN, Song DD, Liu WW, Ling C, Wu FX (2023). TRAF3/STAT6 axis regulates macrophage polarization and tumor progression. Cell death and differentiation.

[24] Zhang R, Shen Y, Zhang Q, Feng X, Liu X, Huo X (2023). TRIM21-mediated Sohlh2 ubiquitination suppresses M2 macrophage polarization and progression of triple-negative breast cancer. Cell death & disease.

[25] Tang PM, Nikolic-Paterson DJ, Lan HY (2019). Macrophages: versatile players in renal inflammation and fibrosis. Nature reviews Nephrology.

[26] Witherel CE, Abebayehu D, Barker TH, Spiller KL (2019). Macrophage and Fibroblast Interactions in Biomaterial-Mediated Fibrosis. Advanced healthcare materials.

[27] Shao M, Cheng H, Li X, Qiu Y, Zhang Y, Chang Y (2024). Abnormal mitochondrial iron metabolism damages alveolar type II epithelial cells involved in bleomycin-induced pulmonary fibrosis. Theranostics.

[28] Zhou Z, Yao J, Wu D, Huang X, Wang Y, Li X (2024). Type 2 cytokine signaling in macrophages protects from cellular senescence and organismal aging. Immunity.

[29] Yu T, Gan S, Zhu Q, Dai D, Li N, Wang H (2019). Modulation of M2 macrophage polarization by the crosstalk between Stat6 and Trim24. Nature communications.

[30] Deng C, Huo M, Chu H, Zhuang X, Deng G, Li W (2024). Exosome circATP8A1 induces macrophage M2 polarization by regulating the miR-1-3p/STAT6 axis to promote gastric cancer progression. Mol Cancer.

[31] Alvarez MM, Liu JC, Trujillo-de Santiago G, Cha BH, Vishwakarma A, Ghaemmaghami AM (2016). Delivery strategies to control inflammatory response: Modulating M1-M2 polarization in tissue engineering applications. Journal of controlled release: official journal of the Controlled Release Society.

[32] Craig VJ, Zhang L, Hagood JS, Owen CA (2015). Matrix metalloproteinases as therapeutic targets for idiopathic pulmonary fibrosis. American journal of respiratory cell and molecular biology.

[33] Barron L, Wynn TA (2011). Fibrosis is regulated by Th2 and Th17 responses and by dynamic interactions between fibroblasts and macrophages. American journal of physiology Gastrointestinal and liver physiology.

[34] Huen SC, Moeckel GW, Cantley LG (2013). Macrophage-specific deletion of transforming growth factor-β1 does not prevent renal fibrosis after severe ischemia-reperfusion or obstructive injury. American journal of physiology Renal physiology.

[35] Rao J, Wang H, Ni M (2022). FSTL1 promotes liver fibrosis by reprogramming macrophage function through modulating the intracellular function of PKM2. Gut.

[36] Zhang L, Wang Y, Wu G, Xiong W, Gu W, Wang CY (2018). Macrophages: friend or foe in idiopathic pulmonary fibrosis?. Respiratory research.

[37] Zhang W, Wan Z, Qu D (2023). Profibrogenic macrophage-targeted delivery of mitochondrial protector via exosome formula for alleviating pulmonary fibrosis. Bioact Mater.

[38] Yang R, Gao N, Chang Q, Meng X, Wang W (2019). The role of IDO, IL-10, and TGF-β in the HCV-associated chronic hepatitis, liver cirrhosis, and hepatocellular carcinoma. Journal of medical virology.

[39] Boutanquoi PM, Burgy O, Beltramo G, Bellaye PS, Dondaine L, Marcion G (2020). TRIM33 prevents pulmonary fibrosis by impairing TGF-β1 signalling. The European respiratory journal.

[40] Enomoto Y, Katsura H, Fujimura T, Ogata A, Baba S, Yamaoka A (2023). Autocrine TGF-β-positive feedback in profibrotic AT2-lineage cells plays a crucial role in non-inflammatory lung fibrogenesis. Nature communications.

[41] Su J, Morgani SM, David CJ, Wang Q, Er EE, Huang YH (2020). TGF-β orchestrates fibrogenic and developmental EMTs via the RAS effector RREB1. Nature.

[42] Huang C, Wang J, Liu H, Huang R, Yan X, Song M (2022). Ketone body β-hydroxybutyrate ameliorates colitis by promoting M2 macrophage polarization through the STAT6-dependent signaling pathway. BMC medicine.

[43] Odegaard JI, Ricardo-Gonzalez RR, Goforth MH, Morel CR, Subramanian V, Mukundan L (2007). Macrophage-specific PPARgamma controls alternative activation and improves insulin resistance. Nature.

[44] Su S, Zhao Q, He C, Huang D, Liu J, Chen F (2015). miR-142-5p and miR-130a-3p are regulated by IL-4 and IL-13 and control profibrogenic macrophage program. Nature communications.

