# Supplementary material for: USP25 stabilizes STAT6 to promote IL-4-induced macrophage M2 polarization and fibrosis
Source: Int J Biol Sci. 2025 Jan 1;21(2):475–89. doi: 10.7150/ijbs.99345 (PMC11705635; doi:10.7150/ijbs.99345)
Supplement: Supplementary file 1 — Supplementary figures and table. [file ijbsv21p0475s1.pdf]

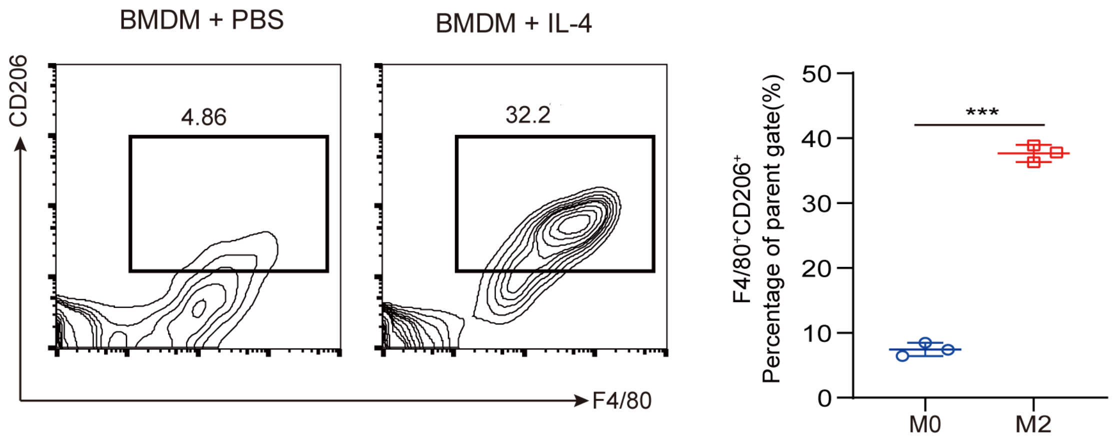

Figure S1.

BMDMs were cultured with M-CSF for 7days and stimulated with 10ng/ml IL-4 or vesicles for 48h to harvest M2 macrophages for flow cytometry.

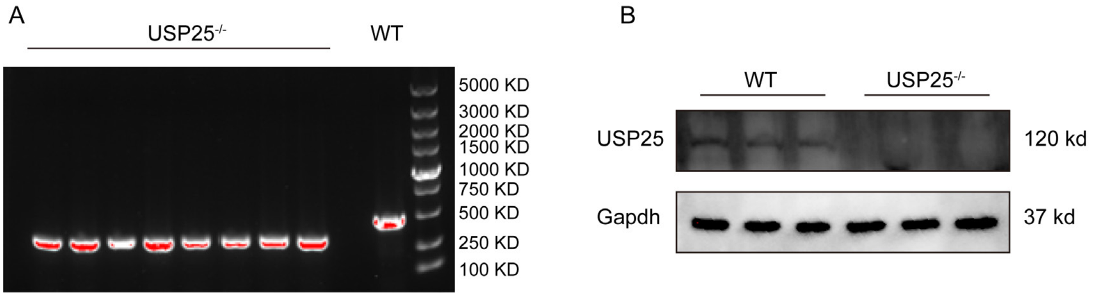

Figure S2.

Identification of USP25 knockout mice.

(A) The USP25 knockout mice. (B) Western Blot Confirmation of USP25 knockout efficiency in mice BMDMs.

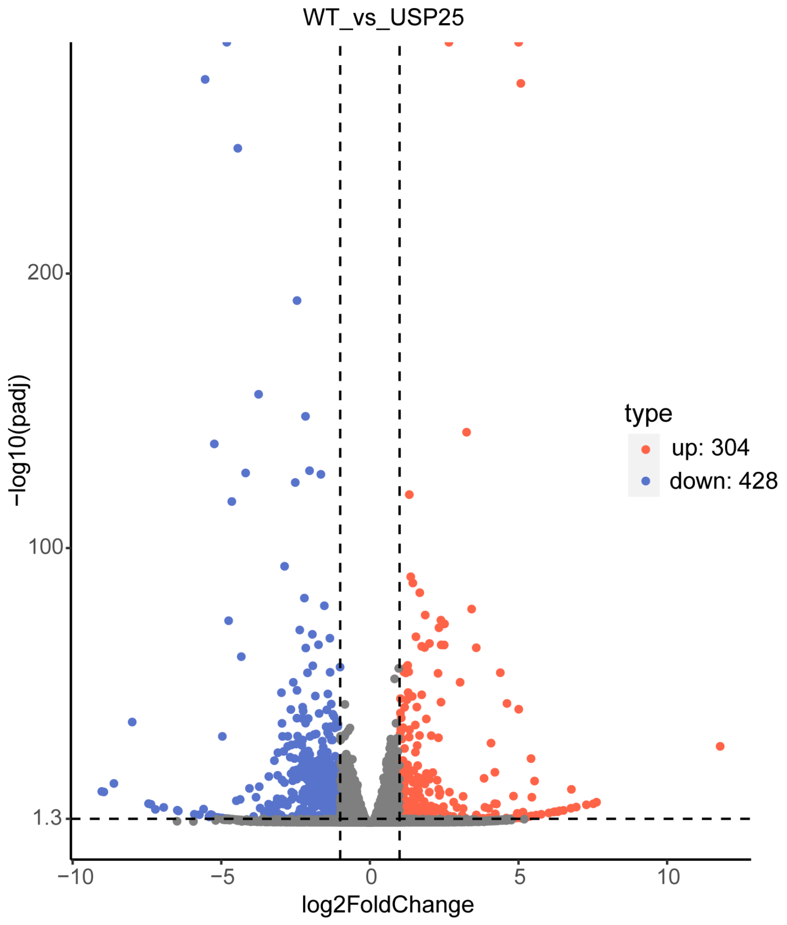

Figure S3. RNA seq analysis results of WT and USP25$^{-/-}$ mice IL-4 stimulated BMDMs. Differential gene volcano map.

Table S1 List of genes and Primers used for qRT-PCR.

Gene Symbol	Primers (5’→3’) (F = forward, R = reverse)
β-actin (mouse)	F: 5’-TTGGGCTCCTGAGGAAACC-3’<br>R: 5’-CGATGTTGTGTAAACCAACCAGA-3’
USP1 (mouse)	F: 5’-ATGCCTGGCGTCATACCTAGT-3’<br>R: 5’-GCAGCTTACTGGGGAGGAC-3’
USP2 (mouse)	F: 5’-TGGGGCTTCTGCTCAACAAAG-3’<br>R: 5’-GAGGCAGTAATCTCTCAGCTCT-3’
USP3 (mouse)	F: 5’-TCAGCCAAGTTCCCCAACG-3’<br>R: 5’-GCCATTCACATACCTTCCACAGT-3’
USP4 (mouse)	F: 5’-AGCACTGCACCTAGCAGAAAT-3’<br>R: 5’-AGACACTGAGCAATAGGGACT-3’
USP5 (mouse)	F: 5’-GTGTTACCGACGATCCGTGTC-3’<br>R: 5’-GTTCATGCAGATATAGAGGCCAC-3’
USP6 (mouse)	F: 5’-TCCGACCAGGATGTAGCACTC-3’<br>R: 5’-CTTCCCAGGGCTCGATCTCT-3’
USP7 (mouse)	F: 5’-AAGTCTCAAGGTTATAGGGACGG-3’<br>R: 5’-CCATGCTTGTCTGGGTATAGTGT-3’
USP8 (mouse)	F: 5’-AGACTCTCCGAAAGCCTTAAACT-3’<br>R: 5’-GCCGTTAATCCTTTGGGTTTTGG-3’
USP10 (mouse)	F: 5’-AACCCACAGTATATCTTTGGCG-3’<br>R: 5’-CCCTCACTAGGTTCGATGACTTC-3’
USP11 (mouse)	F: 5’-AACAACATACCGGACGAGGAT-3’<br>R: 5’-CCTTCATGCCTAGAGGGTTCC-3’
USP12 (mouse)	F: 5’-ACAGTCTCCAAATTCGCCTCC-3’<br>R: 5’-ACTGAGTTGCAGTAGCAGGTATT-3’
USP14 (mouse)	F: 5’-ATGCCACTCTACTCTGTTACAGT-3’<br>R: 5’-AACACCATTGGAGGTTCATCAG-3’
USP15 (mouse)	F: 5’-CCGTGGATGAAAACCTGAGTAG-3’<br>R: 5’-TTCTCTTAGGCAGACAGGGATAA-3’
USP16 (mouse)	F: 5’-AGAAACGGACCAAGGGGAGAA-3’<br>R: 5’-AGAGCCAAACCGAAGGGTCT-3’
USP17 (mouse)	F: 5’-CCCTCAGGATACGTCACCCAT-3’<br>R: 5’-AGGAAGGGATCGAAGGTATGTG-3’
USP18 (mouse)	F: 5’-TTGGGCTCCTGAGGAAACC-3’<br>R: 5’-CGATGTTGTGTAAACCAACCAGA-3’
USP19 (mouse)	F: 5’-TGAACCAGAGCAGTGTACGTT-3’<br>R: 5’-CTGCACCTCGTGTAGCAGG-3’
USP22 (mouse)	F: 5’-CTCCCCACACATTCCATACAAG-3’<br>R: 5’-TGGAGCCCACCCGTAAAGA-3’
USP24 (mouse)	F: 5’-TGGAAAGCCGCGTTTTGACT-3’<br>R: 5’-AGTACCCCACTTGTGAACAGC-3’
USP25 (mouse)	F: 5’-CAGAAGCACCAGCAGACATTT-3’<br>R: 5’-TGGCATTCTTTGCAGTGAGGA-3’
USP26 (mouse)	F: 5’-CTCAAGTCCAGATGTGGAGTGC-3’<br>R: 5’-CTGGTCTTCGCCATAGGTTTG-3’
USP27 (mouse)	F: 5’-GGCAGGAATTGCGGGTTATCT-3’<br>R: 5’-CACGACTTTGCCTTGCTTTTC-3’
USP28 (mouse)	F: 5’-GGGTCCGAGAAGGAAAGCC-3’<br>R: 5’-CACGGAACGATCCGAAGGAAG-3’
USP29 (mouse)	F: 5’-TCCGCAGCACAAACAGGAG-3’<br>R: 5’-CTCACCACTAACCACTACGCC-3’
USP30 (mouse)	F: 5’-CCTCCACTTTGCGAGCCTC-3’<br>R: 5’- CAATCCCACCGATCACTCCC-3’
USP31 (mouse)	F: 5’-GAGCAAAGCAGATTCTTCCAGG-3’<br>R: 5’-CTCAAGCTCCGGTCAGAAGTC-3’

USP32 (mouse)	F: 5’-TTCCCTCAGGACAACCAAAAAG-3’<br>R: 5’-GCAATCTGTTTGAATTGGACTGC-3’
USP33 (mouse)	F: 5’-GAGGTTTGTTGTCTCATGTGTCC-3’<br>R: 5’-GTTCATCTCTGGCGAAGAAGG-3’
USP34 (mouse)	F: 5’-GATATTGGTGGTCGTTCATGTGT-3’<br>R: 5’-TTGGCAAATTCGTAAAGGAAAGC-3’
USP36 (mouse)	F: 5’-CAGCCTCTGCCAAGAAGGTG-3’<br>R: 5’-CCACTTCCAGACACACGCT-3’
USP37 (mouse)	F: 5’-AGTCAGCCTGCTCGTTCACTA-3’<br>R: 5’-AAGTCAACATTAGGCGGCTTT-3’
USP38 (mouse)	F: 5’-GTCGCCATCCTGGACTACATT-3’<br>R: 5’-CTCTCGCAAACCATTCGCAG-3’
USP40 (mouse)	F: 5’-AAATCAGGGTGGAACCTGTTACC-3’<br>R: 5’-GAAGCTGTCGGTGAGGTCT-3’
USP42 (mouse)	F: 5’-TAAACACCGGGATCGAGAAAGC-3’<br>R: 5’-GCATCAGAGTATGCTCCTGAAAA-3’
USP45 (mouse)	F: 5’-CAGACCAAGATCATAACAAGGGC-3’<br>R: 5’-GTCGTTAGGTGAGGAGTGCTT-3’
USP46 (mouse)	F: 5’-ATGACTGTCCGAAACATCGCC-3’<br>R: 5’-TTGACCAATCCGAAGTAGTGTTC-3’
USP47 (mouse)	F: 5’-CGCGTCCGCTGAATGTTTG-3’<br>R: 5’-TGTCACACGATGTCCCGTCT-3’
USP48 (mouse)	F: 5’-CAGAGGAAACCCGAATTGCTT-3’<br>R: 5’-GTGGCTCCCAGGTTAGTCAAG-3’
USP49 (mouse)	F: 5’-AGTTCCGGGAATGTTTCCTGA-3’<br>R: 5’-CTCCTTACTGACAACTCTGCG-3’
USP52 (mouse)	F: 5’-TAGCTGATCGGAAGGAACTGA-3’<br>R: 5’-ACCCCACTCATGCTCGTCA-3’
USP53 (mouse)	F: 5’-AAGGCATGAGCGAGTTAAACC-3’<br>R: 5’-AAGCAACTGTGATACCCCAGG-3’
USP54 (mouse)	F: 5’-TTCTGGAAGCCGAGGTAGTGT-3’<br>R: 5’-GGCGGAAGATGTCCAAGTG-3’
α-SMA (mouse)	F: 5’-GGCTTCGCTGGTGATGATGCTC-3’<br>R: 5’- TCCCTCTCTTGCTCTGGGCTTC-3’
Collagen I<br>(mouse)	F: 5’-GCTCAGAGGCGAAGGCAACAG-3’<br>R: 5’-GATGGGCAGGCGGGAGGTC-3’
Tgfbr1 (mouse)	F: 5’-CTCCCGTGGCTTCTAGTGC-3’<br>R: 5’-GCCTTAGTTTGGACAGGATCTG-3’
Tgfbr2 (mouse)	F: 5’-TTGGATTGCCAGTGCTAACCC-3’<br>R: 5’-AACAAGCCACAGTAACATGACA-3’
TGF-β (mouse)	F: 5’-ACTGGAGTTGTACGGCAGTG-3’<br>R: 5’-GGGGCTGATCCCGTTGATTT-3’
VEGF (mouse)	F: 5’-TATTCAGCGGACTCACCAGC-3<br>R: 5’-AACCAACCTCCTCAAACCGT-3’
Arg 1 (mouse)	F: 5’-ACATTGGCTTGCGAGACGTA-3’<br>R: 5’-ATCACCTTGCCAATCCCCAG-3’
YM1 (mouse)	F: 5’-CAGGTCTGGCAATTCTTCTGAA-3’<br>R: 5’-GTCTTGCTCATGTGTGTAAGTGA-3’
Fizz1 (mouse)	F: 5’-CCTGCTGGGATGACTGCTAC-3’<br>R: 5’-CAGTGGTCCAGTCAACGAGT-3’
IL-6 (mouse)	F: 5’-TAGTCCTTCCTACCCCAATTTCC-3’<br>R: 5’-TTGGTCCTTAGCCACTCCTTC-3’
